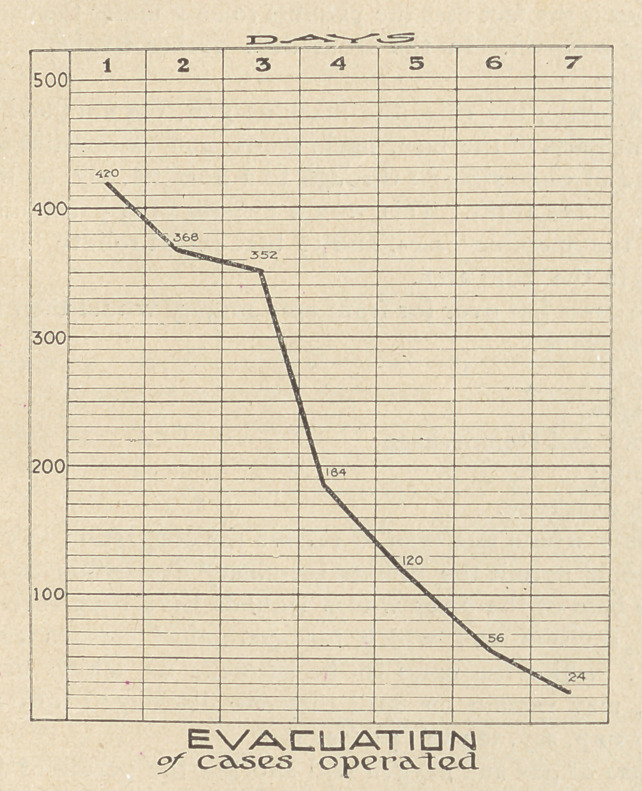# Treatment of 420 Infected Wounds under Battle Conditions Arriving on the Average of 58 1/3 Hours after Injury without Previous Surgical Treatment

**Published:** 1918-11

**Authors:** G. W. Crile


### RESEARCH COMMITTEE REPORT

**Treatment of 420 Infected Wounds Under Battle Conditions Arriving on the Average of 58 1/3 Hours After Injury Without Previous Surgical Treatment**[1]. By the Surgical Staff of the Lake-side Unit.

Reported by LIEUTENANT-COLONEL G. W. CRILE, as a part of the work of the Sub-Committee on Infected Wounds appointed by the Research Committee of the American Red Cross in France. Presented at the September and October meetings.

### *Introduction.*

This is a report of a " group research " by the Lakeside Unit on the treatment of infected wounds at a base hospital during a battle. This work was made possible through the necessary authorization by General Burtchaell; the co-operation of General Bowlby; the advice of Colonel Gask and Colonel Elder; and the co-operation of the D. D. M. S., Colonel Meek. The surgical work was performed by Captain Rogers, Captain Graham, Captain Barney, Captain Brock, Lieutenant Harrison, Lieutenant Jackson, Lieutenant Thompson, Lieutenant Sherry, Lieutenant Hinton and Lieutenant Meader; the pathologic work by Lieutenant Richardson.

*The Problem.*

In this series the patients were on the operating table for their first surgical treatment on an average of 58 1/3 hours after injury — the shortest period being 24 hours, the longest 150 hours. As it developed, our problem was this : what can be done to reclaim the infected wounds, inclusive of the head and chest and abdomen, arriving for the first surgical treatment on the 58 1/3 hour after injury? To be useful in the military sense, any plan must be limited to the equipment and personnel of a standard military hospital; it must be an every-day method; and must be adapted to battle conditions. At the 58th hour, the wound presented heat, swelling, tenderness, redness, and a discharge of purulent fluid; in this series, the wounds had been surgically untouched, undrained, not packed, but dressed and left to natural defenses up to the time of arrival.

*Surgical Management.*

1. There were four operating tables at which

2. Two surgeons operated during eight-hour periods, leaving time for each surgeon personally to supervise the after-care of his patients, to keep his records, to secure rest, and to return refreshed for his next turn at the table.

3. The daily turn-over was just over 100 operations during the acute period.

4. Every patient was examined on the table by his surgeon before anesthesia.

5. All operations were performed on the ordinary ambulance stretcher.

6. Nurse assistants and nurse anesthetists were employed.

7. All operative cases were prepared under anesthesia by :

*a*) Scrubbing thoroughly a wide field with soap and water;

*b*) Shaving;

*c*) 5 o/o Sodium Carbonate;

*d*) Ether;

*e*) Alcohol.

(Captain Barney recently introduced sodium carbonate as a supplement to soap and scrubbing.)

8. The limb and the wounds were handled with the same care during anesthesia as before anesthesia.

9. Every wound that had not progressed to abcess and new tissue formation was treated by complete surgical revision.

10. In large wounds no attempt was made to excise *en bloc* within the line of uninfected tissue, but the devitalized tissue was dealt with in an opportunist manner.

11. But little skin was excised.

12. Ample exposure was always made, usually by vertical incisions.

13. After surgically meeting the indication for today — namely, complete revisions — the " tomorrow " of the wound was considered, and certain cases were treated by

14. Incision of fascia overlying swollen muscles; incision of the skin and superficial fascia, placed where it seemed certain that within the first and second post-operative days swelling and tension would appear; that is, the operation that was anticipated as being necessary day after tomorrow was done now, but, owing to these anticipatory or prophylactic incisions, little or no swelling occurred later. In other words, the surgeon aimed to take and to hold the initiative.

15. When completed, the wound was flat and soft and anticipatory — the obstacle to its biologic defense had been removed. When the wound was surgically completed, it contained many bacteria. The bacteria were facing not the normal antagonism of living tissue, but the heightened antagonism of tissue whose emergency defense had been called out by the adequate stimulus of the injury and the bacteria. The wound was in the state of a heightened defense clinically so well known in the two-stage operation in civil surgery. There was active phagocytosis and increased blood supply everywhere, and the infecting bacteria had not gained virulence by selective struggle against antiseptics — a principle established for protozoa and recognized clinically for infection.

Having completed the revision, what shall be done with the wound, taking into account battle conditions?

We tested five plans each applicable to such periods, each surgeon dividing his cases among the five plans as follows :

1. Surgery, plus dry gauze dressing — no antiseptics.
2. Flavine.
3. Dichloramin-T chlorcosane.
4. Wright's hypertonic pack.
5. Alcohol.

Although recognizing the sterling merit of Carrel-Dakin, we did not use it because we were testing only those methods available for rush periods. We did not use Bip because we hoped in suitable cases to do delayed primary suturing.

*General Statement and Clinical Results*

1. In the period there were admitted 1274 patients.
2. Of these 660 were walkers, and 614 were stretchers.
3. Of the 614 stretcher cases, 194 required no operation because they were taking care of themselves; and 420 required operation.
4. Of the 420 operative cases, 67 were marked for delayed primary suture; 44 superficial wounds were immediately sutured.
5. Of the 44 immediate sutures, all were successful; of the successful cases, there were two fractured humeri, and one fractured radius.
6. Of the 67 delayed primary sutures, 91 per cent. healed without requiring removal of any stitches; 6.1 per cent. were partial successes; 2.9 per cent were failures.
7. There were 95 compound fractures.
8. Among 420 operative cases, there were four, or 0.9 per cent. deaths.
9. There was no case of bacteriemia or septicemia.
10. No case was rejected for operation because it was thought that the infection had gone too far.
11. The patients not suitable for delayed suture were quickly ready for evacuation, as seen by the chart.
12. The comparative results in the various types of treatment

COMPOSITE TEMPERATURE CHARTS

420 operations are well indicated by the charts showing composite temperature curve of the cases treated by :

*a*) Plain surgery — dry gauze — no antiseptics.

*b*) Dichloramin-T chlorcosane.

*c*) Flavine.

*d*) Wright's hypertonic pack.

*e*) From the foregoing it would appear that surgical revision of wounds untreated for 58 1/3 hours causes no harm and does much good. It is felt that with further experience a greater percentage of delayed infected wounds, including compound fractures, will be closed both primarily and delayed: the mechanism by which this benefit follows will be further discussed later; that the presence or the absence of chemical antiseptics made no notable impression on the clinical course after operation: this point will be considered further. These results are a tribute to the judgment of the surgeons in the forward area who selected the cases for evacuation without operation, and who selected to pack no gauze and insert no drainage tube into those wounds, but merely dressed them.

*Bearing of the Results of Delayed Treatment on Battle Surgery.*

Since in this series we were dealing with infected wounds, averaging 58 1/3 hours; since in no instance did we see develop, either in operated or in unoperated cases, a case of bacteremia; and since the wounds of the type we dealt with were so satisfactorily reclaimed, it would seem to indicate that for a considerable percentage of wounds a base hospital may serve the purpose of a forward hospital. For it is apparent that the delay in our series did not materially alter the mortality nor the morbidity. Certain administrative advantages are obvious, and although there are certain clinical disadvantages, there are also clinical advantages. Among the disadvantages are the advancing infections; among the advantages is the fact that the patients remain under the care of the operating surgeon. In the front area during battles this is not practicable.

*a*) In a battle, the rifle, the machine gun, the shrapnel wounds of the soft parts of the face, neck, trunk, and extremities might be evacuated at once straight through to a hospital, say, up to within 75 miles of the front. This would dispose of a considerable per cent. of the operable cases, and to that extent relieve the surgical facilities in the front area.

*b*) The cases left over for front area surgery would then be :

1. Abdomens;
2. Chests;
3. Thighs;
4. Shock and Hemorrhage;
5. Joints;
6. Gas gangrene.

Not only would this tend to relieve the congestion at the front, but it would permit the prompt secondary evacuation back to the ultimate base of the cases whose wounds have been reclaimed by surgical revision and which for anatomical reasons cannot be sutured : while the cases to be sutured, or which have been sutured, may remain on until they are discharged into a convalescent camp, say, ten to 14 days.

The use of gas and oxygen will shorten the period of detention and speed evacuation.

A continuous diffused evacuation will put numerous surgeons at work simultaneously; will diminish the period of waiting for treatment; will afford a greater service to a greater number, instead of ideal service to a minority.

From the favorable results in immediate and delayed primary suture in these cases, it would seem desirable to make an attempt to close a greater number of wounds. The advantage of closure over healing by cicatrization is so great that this subject deserves our greatest consideration.

*Delayed Primary Closure in Infected War Wounds.*

The following plan suggested by Captain Rogers worked out well during periods of rush for making primary and delayed primary wound closures. Lieutenant Hinton was put in charge of the wound closures. Wards were set apart; a sterile operating room established; a head nurse was appointed and such additional assistants as needed. It amounted to a hospital within a hospital.

*Treatment Preparative for Wound Closure.*

1. Certain superficial wounds without fever or local inflammation are treated by immediate excision and suture. If there is a dusky red epithelial edge or surface discharge, methyl-alcohol dressings are applied twice a day until the wound is dry. Alcohol chars the surface, leaving a covering resembling a scab.

2. If there is acute inflammatory reaction and fever, hot dressings are applied. These consist of gauze and lint wrung out in boiling water and applied as hot as the patient can stand them. The part is then wrapped in jaconnet. The dressings should extend wide of the wound on both sides. When the temperature falls, the inflammation subsides, and discharge diminishes, methyl alcohol twice a day is used as above described. Hot dressings are put on every four hours during the day and alcohol dressings at night; the alcohol dressings are removed in the morning and hot dressings resumed. This prevents the maceration of the skin. Hot dressings should be discontinued as early as possible in order not to lower the skin's resistance. By these methods a wound should be ready for excision and suture in from three to five days, providing there is no streptococcus infection. Four hundred and fifty light wounds were treated in this manner. In 95 per cent. of these there was primary healing.

*Delayed Primary Suture.* (In this series the debridement of some wounds was made at the C. C. S. : the remainder at the hospital.) If there is no fever, odor, or swelling, the dressings next to the skin are not removed until operation. If for any reason the wound dressing is removed before the time of closure, hot dressings should be applied for at least one day to develop a local reaction and re-establish the normal defense. In compound fractures, hot dressings are used until the day of operation.

The wounds debrided at the front and dressed with gauze soaked in flavine have come to the base in the best condition. Wounds dressed with dry sterile gauze have done well. An objection to dry gauze is that its removal is difficult and painful.

*Time for Operation.*

1. When the wound treated by hot dressings, alternating with alcohol, is dry and charred, and no inflammatory line is seen, the wound is sterile and may be excised and immediately sutured.

2. The third day after operation is probably the best time to close a case of delayed primary suture. Before that time the wound resistance has not been fully established, and after that time the epithelium begins to grow or infection occurs.

Fever is not necessarily a contra-indication to wound closure, for it may be due to other causes.

3. The presence of bacteria of itself is not a contra-indication, for more than one-half of the successful closures would have been excluded on laboratory findings. On clinical grounds, no wound having hemolytic streptococci was closed; but anaerobes including B. Tetanus, Welchii, Sporogenes, and aerobes including Pyocyaneus and non-hemolytic streptococci have been closed in wounds with success. It is unwise to suture wounds in very ill patients.

*Preparation of Field of Operation.*

The actual wound dressing, if present, is left *in situ*, the skin scrubbed with soap and water, shaved, and washed with sodium carbonate solution, ether, alcohol and iodine. Ether may be omitted. The dressing is then removed and culture taken.

*Points in the Technique of Wound Closure.*

1. It is necessary to make certain that no dead space is left anywhere in the wound; especially in its deeper recesses.

2. Sutures should be tied loosely: tension tends to defeat healing.

3. When possible, pass the sutures entirely around the wound, and not through it.

4. Make certain that no oozing occurs after the wound is closed by making steady pressure on the wound for a few minutes after all the stitches but one are tied; then, when every drop of fluid is pressed out through the hiatus of the last untied stitch, this suture is tied.

5. In doubtful cases healing is promoted by hot dressings.

6. The status of the wound, as to its fitness for closure, may be accurately estimated by clinical signs alone.

## Figures and Tables

**Figure f1:**
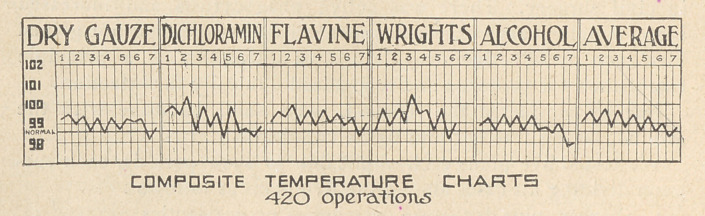


**Figure f2:**